# Development and psychometric evaluation of the Group Home Culture Scale

**DOI:** 10.1111/jar.12693

**Published:** 2019-12-23

**Authors:** Lincoln Humphreys, Christine Bigby, Teresa Iacono, Emma Bould

**Affiliations:** ^1^ Living with Disability Research Centre La Trobe University Bundoora Victoria Australia; ^2^ Department of Occupational Therapy Monash University Melbourne Victoria Australia

**Keywords:** group home, intellectual disability, organizational culture, service delivery, supported accommodation

## Abstract

**Background:**

Organizational culture in group homes for people with intellectual disabilities has been identified as influencing staff behaviour and residents’ quality of life (QOL). Despite this influence, culture has been under‐researched, with no published and validated instrument to measure its dimensions in group homes. The aim was to develop such a measure.

**Method:**

The Group Home Culture Scale (GHCS) was developed using a theory‐driven approach. Items were generated from the research literature, which were reviewed by experts and tested in cognitive interviews. Data from 343 front‐line staff were used for exploratory factor analysis.

**Results:**

The content and face validity of the GHCS were found to be acceptable. Exploratory factor analysis indicated that the GHCS measured seven dimensions of group home culture. Cronbach's alpha for the dimensions ranged from 0.81 to 0.92.

**Conclusions:**

The GHCS has potential use in research to determine whether dimensions of group home culture predict the quality of staff support and residents’ QOL.

## 1 INTRODUCTION

Since the implementation of deinstitutionalization policy in Australia and other countries, such as England, Sweden and the United States, group homes have become a main form of supported accommodation for adults with intellectual disabilities (Larson, Ryan, Salmi, Smith, & Wuorio, 2012; Mansell, Beadle‐Brown, & Special Interest Research Group, 2010; Tøssebro et al., 2012). Quality of life (QOL) has often been measured as an indicator of service quality in studies of supported accommodation services (Walsh et al., 2007). Schalock, Verdugo, Gomez, and Reinders (2015) defined QOL as a “multidimensional phenomenon composed of core domains that constitute personal well‐being” (p. 2). Overall, research evidence has shown that people with intellectual disabilities experience better QOL living in group homes compared to institutions (Bigby, Cooper, & Reid, 2012; Emerson & Hatton, 1996; Kozma, Mansell, & Beadle‐Brown, 2009). Research has also shown that there can be variability in the QOL of people living in group homes (Mansell, Beadle‐Brown, & Bigby, 2013).

Researchers have identified a range of variables to account for this variability in QOL outcomes and service quality (Bigby & Beadle‐Brown, 2018; Felce & Perry, 2007). These variables include (a) size and location of group homes (Felce, 1998); (b) resources, such as financial and staff‐resident ratios (Felce, 2017); staff characteristics, such as qualifications (Mansell, Beadle‐Brown, Whelton, Beckett, & Hutchinson, 2008); (d) staff practices, such as active support (Mansell & Beadle‐Brown, 2012); and (e) front‐line management, such as practice leadership (Beadle‐Brown, Bigby, & Bould, 2015; Bigby, Bould, Iacono, Kavanagh, & Beadle‐Brown, 2019). Nevertheless, the predictors of high‐quality services and good QOL remain incompletely understood (Bigby & Beadle‐Brown, 2018; Felce & Perry, 2007). Adequate resources and settings of small size, for example, have been proposed to be necessary but not sufficient conditions for good outcomes (Bigby & Beadle‐Brown, 2018; Emerson & Hatton, 1996). It has been suggested that organizational factors, such as how resources are used (Stancliffe, Emerson, & Lakin, 2004), management practices (Bigby & Beadle‐Brown, 2018; Bigby, Bould, Iacono, & Beadle‐Brown, 2019), and the way staff support is delivered and monitored may be critical (Emerson & Hatton, 1996). Organizational culture has been consistently identified as influencing service quality (Felce, Lowe, & Jones, 2002; Hastings, Remington, & Hatton, 1995; Mansell & Beadle‐Brown, 2012; Walsh et al., 2010) based on the assumption that culture influences staff behaviour, and in turn, residents’ QOL.

The notion that culture influences staff behaviour has been evident in the wider organizational literature since the concept came to prominence in the 1980s (Ehrhart, Schneider, & Macey, 2014; Smircich, 1983). Many definitions of organizational culture have appeared in the literature (Verbeke, Volgering, & Hessels, 1998). For example, it has been broadly defined as “the way we do things around here” (Deal & Kennedy, 1982, p. 4) and more comprehensively defined by Schein (2010) as

…a pattern of shared basic assumptions learned by a group as it solved its problems of external adaptation and internal integration, which has worked well enough to be considered valid and, therefore, to be taught to new members as the correct way to perceive, think, and feel in relation to those problems. (p. 18)

Culture has also been conceptualized as existing at multiple levels, for example at the broader organizational and staff group (or team) levels, with potentially numerous subcultures within organizations (Martin, 2002; Trice & Beyer, 1993). Despite differing conceptualizations, key concepts of culture include shared beliefs, values, norms, assumptions, ideologies and meanings (Alvesson, 2013; Ehrhart et al., 2014).

The conceptualization of culture frequently discussed in the intellectual disability services literature has included a distinction between formal and informal organizational culture (Emerson, Hastings, & McGill, 1994; Felce et al., 2002; Hastings et al., 1995; Mansell & Beadle‐Brown, 2012). Formal culture refers to operational policies, job descriptions, planning systems, working methods, training and mechanisms used to monitor staff (Felce et al., 2002). Arguably, they are aspects that are controlled by management and designed to influence staff behaviour through specifying expectations and constraining what staff do (Felce et al., 2002). Informal culture, on the other hand, refers to ways of working as defined by the staff group, interactions and relationships among staff, and also between staff and residents, and reasons for working (Felce et al., 2002; Hastings et al., 1995). For more than 20 years, congruence between formal and informal culture has been argued to contribute to higher quality services than incongruence (Emerson et al., 1994), but research has not been conducted to confirm this proposition.

There have been few studies into organizational culture in group homes, or more generally supported accommodation services. In one such study, Gillett and Stenfert‐Kroese (2003) examined organizational culture and residents’ QOL in two residential units from the same organization using the Organizational Culture Inventory (OCI; Cooke & Lafferty, 1989, as cited in Gillett & Stenfert‐Kroese, 2003). They found that residents in the unit with the more positive culture also had higher QOL. The OCI is a generic instrument that measures organizational culture in terms of behavioural norms (Cooke & Szumal, 2000), and has been used by researchers in a wide variety of organizations (Kummerow & Kirby, 2014). It comprises 120 items, and although some appear applicable to supported accommodation services (e.g. staff members “help others to grow and develop” and “do what is expected”), others appear less relevant (e.g. staff members “turn the job into a contest” and “use the authority of their position”; Balthazard, Cooke, & Potter, 2006, p. 720).

In another study applying a generic measure, Hatton et al. (1999) used the Organizational Culture Profile (OCP; O'Reilly, Chatman, & Caldwell, 1991) to examine associations between culture and staff outcomes (e.g. job satisfaction, commitment) in services for people with intellectual disabilities. The OCP assesses values through 54 statements. Although some statements arguably are relevant to supported accommodation services (e.g. “being people oriented” and “enthusiasm for the job”), some are less so (e.g. “high pay for good performance”, “being competitive” and “being aggressive”; O'Reilly et al., 1991, p. 516).

These two generic measures, the OCI and OCP, were not designed for group homes. As such, they do not measure culture relating to interactions between staff and residents of group homes and are mostly about interactions among staff and employment conditions.

The limited research into culture in group homes for people with intellectual disabilities has primarily used qualitative methodologies. Using an ethnographic approach, and Schein’s (2010) definition of culture, Bigby and colleagues (Bigby & Beadle‐Brown, 2016; Bigby, Knox, Beadle‐Brown, & Clement, 2015; Bigby, Knox, Beadle‐Brown, Clement, & Mansell, 2012) conducted two studies in five underperforming and three better performing group homes for people with severe intellectual disabilities. They identified five dimensions of culture: (a) alignment of power holders’ values, (b) regard for residents, (c) perceived purpose, (d) working practices and (e) orientation to change and new ideas. Each dimension was proposed as a continuum from negative, which was more likely to apply to underperforming group homes, to positive, which was more likely to apply to high performing group homes.

Comparisons of the culture across the underperforming and better performing group homes in these studies demonstrated that the way staff worked and interacted with the residents were markedly different. For instance, staff practices in the underperforming group homes were characterized as staff‐centred, prioritizing completion of tasks and interactions that involved staff doing things for or to residents. In contrast, staff practices in the better performing group homes were characterized as person‐centred, whereby relationships between staff and residents were described as warm, with interactions that included moments of fun. Furthermore, Bigby, Knox, Beadle‐Brown, and Bould (2014) showed that the residents who lived in the group homes with more positive cultures had higher QOL. Bigby and colleagues (Bigby & Beadle‐Brown, 2016; Bigby, Knox, et al., 2012) suggested that the dimensions they identified could provide a basis to develop a quantitative measure of organizational culture in group homes, with potential advantages over the generic instruments used to date. They further argued that such an instrument would be more likely to point to relevant implications for service delivery, and be more translatable into strategies to improve culture than generic instruments.

This study aimed to develop the type of instrument for measuring culture in group homes suggested by Bigby and colleagues (Bigby & Beadle‐Brown, 2016; Bigby, Knox, et al., 2012). The definition of organizational culture used required concepts that could be more readily measured using quantitative methods, than those in Schein’s (2010) definition, such as staff members’ assumptions, which require intensive observations and interviews to be identified. Accordingly, culture was defined using features described across the broad organizational culture literature, and then those pertinent to group homes for people with intellectual disability, as staff members’ shared values, beliefs, norms and patterns of behaviour that influence how they think, feel and act (Ott, 1989; Trice & Beyer, 1993). The purpose of this study was to develop and evaluate the psychometric properties of an instrument to measure dimensions of organizational culture in group homes—named the Group Home Culture Scale (GHCS). The GHCS was designed as a self‐report instrument to be completed by disability support workers (DSWs) and front‐line supervisors, who are variously known as house supervisors, team leaders or service managers.

## 2 METHOD

### 2.1 Design

A mixed‐methods sequential research design (Creswell & Plano Clark, 2011) was used with the following stages (a) item development, (b) expert review, (c) cognitive interviews and (d) questionnaire administration. Quantitative and qualitative data were collected and analysed to refine the conceptualization and measurement of constructs, as well as to assess the content validity, face validity and internal consistency of the GHCS. Ethics approval for this study was provided by the La Trobe University Human Research Ethics Committee.

### 2.2 Item development

Development of the GHCS was theory‐driven (DeVellis, 2012; Hinkin, 1998; Wymer & Alves, 2013), based on the five dimensions of group home culture proposed by Bigby and colleagues (Bigby & Beadle‐Brown, 2016; Bigby, Knox, et al., 2012), and research examining culture in supported accommodation services where allegations of staff abuse has occurred (Cambridge, 1999; Marsland, Oakes, & White, 2007). Drawing on this literature, the content domain of each dimension was specified (i.e. the concepts and elements that comprised each dimension) and then items were written to tap each dimension (DeVellis, 2012). Items were written to tap both ends of the continuum (i.e. positive and negative) for each dimension, and to adhere to item writing guidelines, such as simple and direct language be used, and each item conveys a single idea (Gideon, 2012). Consistent with the referent shift consensus composition model, items were phrased so that respondents reflected on their staff group (or team) and their shared, rather than individual ways of working (Chan, 1998; Klein, Conn, Smith, & Sorra, 2001). For example, the statement “Staff plan with residents what happens on weekends” has a group referent, in contrast to “I plan with residents what happens on weekends,” which has an individual referent.

A large pool of items (*n* = 359) was generated by the first author, which was reviewed and refined by the research team over several meetings. Items that tapped the dimensions were identified, others were either revised or discarded, and new items were generated. Following this process, 197 items were developed (range = 32–50 items per dimension).

### 2.3 Expert reviews

The initial 197 items were reviewed by experts, defined as academics who had published research on supported accommodation services. Twelve experts, identified using purposive sampling, were sent information via email about the study and invited to participate. Those who expressed interest were sent further information, including the definition of organizational culture, conceptual descriptions for each dimension, and the list of items which they were asked to rate on a 4‐point Likert scale (1 = not representative to 4 = completely representative) according to the extent to which each item measured the dimension and its clarity (1 = not clear to 4 = very clear). Written feedback was also sought regarding the clarity of the items, suggestions for improved phrasing or new items, whether any items should be deleted or moved to another dimension, and comments on the conceptualization of the dimensions.

Four reviews were returned. Two experts rated all the items, one rated the representativeness for most (82%), and another rated the items inconsistently, but provided comments for many. The Content Validity Index (CVI; Lynn, 1986; Polit & Beck, 2006) was used to analyse the representativeness ratings for each item by calculating the proportion of experts that assigned it a rating of 3 or 4. Lynn (1986) recommended a CVI of 1.0 to retain an item when there are five or fewer participants. Using the ratings from three experts resulted in 82 items obtaining a CVI of 1.0 (CVI range = 0.33–1.0). The CVI was then calculated for items rated by only two experts, which resulted in an additional 30 items obtaining a CVI of 1.0. However, there were two problems with using only the CVI to retain items: (a) the retained items may not have tapped the content domains of the dimensions comprehensively; and (b) the qualitative data were not considered. To address these problems, the retained items were mapped according to the content domains for each dimension. For those content domains that were not comprehensively tapped, the suitability of the items with a CVI of less than 1.0 was assessed. This process involved tallying the representativeness ratings provided by three experts (range = 3–12), and items with a total score of 9 or 10 were assessed as to whether they should be retained based on a review of the written comments and clarity ratings. Eleven items were retained using this process, some of which were then revised.

Over several meetings, the retained items (*n* = 123) were reviewed by the research team, and the comments and suggestions made by experts were discussed. Following this process, 41 new or revised items were added, bringing the total to 164 items.

### 2.4 Cognitive interviews

To test the items, 16 cognitive interviews (Willis, 2005) were conducted with 15 participants who had experience of working in group homes, recruited using convenience and snowball sampling. Once they provided written consent, they were emailed a questionnaire that comprised the items and asked to complete it either before or during the interview. Telephone interviews were conducted, most of which were approximately 1 hr in duration (range = 20–80 min), and handwritten notes were made. One participant who had extensive experience as a disability support worker was interviewed twice, in separate rounds, because he provided significant insight into the cognitive process of interpreting and answering items. Participants were asked how they understood and interpreted items, how they formulated answers, and to describe the experiences they recalled to answer questions. They were also asked about the design of the questionnaire, such as the layout, instructions and the response format.

Following Willis’ (2005) recommendation, interviews were conducted in rounds, with data analysed between them, until all major problems had been corrected. After each round, data were analysed by producing summaries and making comparisons across participants (Miller, Willson, Chepp, & Ryan, 2014). Items that were consistently understood as intended were retained; items that were problematic or ambiguous were either dropped from the questionnaire, or revised and retested in subsequent interviews. In this way, the face validity of the GHCS was assessed. After four rounds of interviews, 86 items remained.

### 2.5 Questionnaire administration for exploratory factor analysis

In order to identify the underlying factor structure and evaluate the internal consistency of the GHCS, a questionnaire comprising the 86 items was administered to DSWs and front‐line supervisors who worked in group homes for people with intellectual disabilities. Nongovernment intellectual disability organizations that operated in Australia were approached via email and managers from 10 organizations agreed to participate in the research. These organizations varied in location and size, operating in three states (New South Wales, Victoria and Western Australia) in both metropolitan and nonmetropolitan areas, and managing from 5 to 31 group homes (*Mdn* = 9).

Inclusion criteria were DSWs and front‐line supervisors who worked in 24 hr staffed group homes in which up to eight adults with intellectual disabilities were supported. Staff were excluded if they had worked in the group homes for <2 months and/or worked, on average, <4 hr per week.

#### 2.5.1 Participants

Questionnaires were completed by 380 staff, representing an overall response rate of 43% (range = 8%–64% across organizations). Of the respondents, 343 (279 DSWs and 64 front‐line supervisors) met the eligibility criteria and provided data that were usable for exploratory factor analysis (EFA).

Participants were on average 44.4 years of age (*SD* = 13.2, range = 20–74), 68.7% were female and 57.4% born in Australia. As shown in Table 1, over half of the participants had more than 3 years’ experience of working in accommodation services. Most participants worked, on average, 26 hr or more per week in the group homes. The mean number of residents per group home was 5.1 (*SD* = 1.1, range = 2–8).

**Table 1:** Education and employment characteristics of staff participants (*N* = 343)

Characteristic	Percent
Education level	
High school	16.0
TAFE certificate 3	8.8
TAFE certificate 4	32.3
Diploma	22.1
University degree	15.4
University post‐graduate	4.8
Other	0.6
Total experience in DAS	
3–6 months	1.5
7–11 months	4.5
1–2 years	11.9
3–5 years	23.2
6–10 years	28.3
11–14 years	10.1
15 years or more	20.5
Experience in group home	
3–6 months	13.5
7–11 months	13.5
1–2 years	24.5
3–5 years	26.9
6–10 years	14.1
11–14 years	4.9
15 years or more	2.8
Hours per week in group home	
5–10 hr	5.5
11–15 hr	4.8
16–20 hr	6.7
21–25 hr	9.7
26–30 hr	13.0
31–35 hr	17.9
36 hr or more	42.4
Employment contract	
Full‐time	48.6
Part‐time	43.8
Casual	7.6

Abbreviations: DAS, Disability Accommodation Services; TAFE, Technical and Further Education.

Totals of percentages are not 100 for every characteristic because of rounding.

### 2.6 Measures

#### 2.6.1 Group Home Culture Scale (GHCS)

Respondents were asked to rate each of the 86 items on a 5‐point Likert scale anchored by strongly disagree (1) and strongly agree (5). The first page contained instructions on how to complete the measure and definitions of key terms (e.g. house supervisor, team, senior managers). Nine items on the DSW and front‐line supervisor versions of the GHCS differed: front‐line supervisors were asked to self‐report about their own leadership, in which case, the referent was themselves; DSWs responded to corresponding items in terms of the leadership of their front‐line supervisor.

#### 2.6.2 Demographic and employment information

Demographic and employment information about respondents was obtained from closed questions (*n* = 13) at the end of the GHCS. These questions addressed gender, age, country of birth, level of education, employment experience in disability accommodation services, and employment experience and average hr per week worked in the group home.

### 2.7 General procedures

Once written consent from a manager at each organization had been obtained, group homes that met the eligibility criterion were identified. Questionnaire packets were posted to the manager or contact person, who then distributed them to front‐line supervisors and DSWs. Completed questionnaires were returned to the research team in provided prepaid envelopes. Data were collected from October 2015 to February 2016.

### 2.8 Analyses

Data were entered and analysed using SPSS 22. Descriptive statistics for the sample were calculated. EFA was conducted for the 86 items GHCS to identify the underlying structure among the variables. EFA was chosen instead of confirmatory factor analysis because the GHCS was a new measure, and although the dimensions and items were developed from theory, the number of factors and their composition were uncertain (Fabrigar, Wegener, MacCallum, & Strahan, 1999).

Prior to EFA, an analysis of missing data was performed, showing missing data for 80 (22.5%) of 356 GHCS questionnaires. Of these questionnaires, those for which there was more than 15% missing data (*n* = 13) were removed from further analysis. Of the remaining 343 questionnaires, there were missing data on 67 (19.5%) for 1–9 items. Expectation maximization was used to impute missing data (Graham, 2009). Negatively phrased items were reverse scored.

The sample of 343 participants exceeded Fabrigar and Wegener’s (2012) suggested minimum size of 200 participants for EFA when communalities range from 0.40 to 0.70, and three or more items load onto each factor. The suitability of the data for EFA was assessed through an examination of the correlation matrix to ensure correlations were of sufficient strength (>0.30), the Kaiser‐Meyer‐Olkin test to determine sampling adequacy and the Bartlett test of sphericity to determine the degree of significant correlations among variables (Hair, Black, Babin, & Anderson, 2014; Tabachnick & Fidell, 2014). Principal axis factoring, which accounts for common variance, was used to extract the factors (Hair et al., 2014). The number of factors to retain was determined by examining eigenvalues and the scree plot, and conducting a parallel analysis. Of these factor retention methods, parallel analysis has been recommended as the most accurate (Zwick & Velicer, 1986). The parallel analysis was performed by comparing the eigenvalues to those obtained from randomly generated datasets based on the same sample size (Pallant, 2013). The number of factors to retain was indicated by the eigenvalues exceeding those that were randomly generated. An oblique (direct oblimin) rotation was performed to allow the factors to correlate. A minimum factor loading of 0.40 on the pattern matrix was used to retain items. The internal consistency of the factors was assessed using Cronbach's alpha. Because Cronbach's alpha has been shown to underestimate the true level of internal consistency when the assumption of tau equivalence is violated (McNeish, 2018), the greatest lower bound (GLB) was also used to assess internal consistency. GLB was calculated using the program JASP 0.9 (JASP Team, 2018).

## 3 RESULTS

Ten items were removed prior to EFA on the basis of weak correlations (<0.30) or multicollinearity (>0.80; Pett, Lackey, & Sullivan, 2003). Factorability for the remaining 76 items was confirmed according to (a) the Kaiser‐Meyer‐Olkin test of sampling adequacy (overall = 0.92, individual items range = 0.77 – 0.96; Hair et al., 2014); and (b) a significant Bartlett test of sphericity *X* ^2^(2,850) = 15,806.36, *p* < .001 (Hair et al., 2014).

Following principal axis factoring, application of the Kaiser criterion indicated that 16 factors be retained. The scree plot did not provide a clear indication of the number of factors to retain. Rather, the parallel analysis indicated that seven factors had eigenvalues exceeding those randomly generated; hence, seven factors were retained and rotated using the direct oblimin method. Finally, following an examination of factor loadings and communalities, 48 items were retained.

The GHCS subscales were named based on an examination of the items with high loadings on each factor (Hair et al., 2014) and with reference to the original conceptualizations of the dimensions (Pett et al., 2003). The seven factors, their names and descriptions are presented in Table 2.

**Table 2:** Descriptions and example items for the Group Home Culture Scale

Factor	Number of items	Description	Example item
1. Supporting well‐being	12	The extent to which staff practices are directed towards enhancing the well‐being of each resident	Staff find ways to involve each resident in their local community
2. Factional	8	The extent to which there are divisions within the staff team that have a detrimental influence on team dynamics	There are distinct groups of staff, rather than one staff team
3. Effective team leadership	5	The extent to which the house supervisor engages in leadership practices that transmits and embeds the culture	The house supervisor role models how to appropriately support and interact with the residents
4. Collaboration within the organization	6	The extent to which staff have a positive perception of organizational support and priorities	Senior managers help us to find solutions to problems
5. Valuing residents and relationships	7	The extent to which staff value the residents and the relationships they have with them	Staff take an interest in the residents’ lives
6. Social distance from residents	5	The extent to which there is social distance between staff and residents, where staff regard the residents to be fundamentally different from themselves	Staff believe that in many ways they are very different to the residents
7. Alignment of staff with organizational values	5	The extent to which staff members’ values align with the espoused values of the organization	As a staff team, our values match the organization's core values

Table 3 provides a summary of the pattern matrix and structure matrix factor loadings and communalities. The majority of the communalities exceeded 0.40, indicating that the variables accounted for an acceptable level of variance (Fabrigar & Wegener, 2012). Cronbach's alpha (also shown in Table 3) ranged from 0.81 to 0.92, indicating very good internal consistency for each factor (DeVellis, 2012). The GLB values (Table 3) ranged from 0.87 to 0.94, providing further evidence of acceptable internal consistency for each factor. The full pattern and structure matrices are presented in the Appendix. Inspection of the pattern matrix (Table A1) showed that none of the retained items cross‐loaded (≥0.40). In total, the seven factors accounted for 55% of the variance. As shown in Table 4, intercorrelations of the factors ranged from −0.36 to 0.47, indicating small to medium correlations across the factors (Cohen, 1992), and that they represent related but also distinct dimensions of group home culture. Table 4 also shows descriptive statistics and sum of squared structure loadings for each factor.

**Table 3:** Summary of factor loadings, communalities, coefficient alphas and the greatest lower bound values for the Group Home Culture Scale

Item	Pattern matrix	Structure matrix	*h* ^2^
Factor 1: Supporting well‐being (*α* = 0.91; GLB = 0.94)			
16. Involve in their local community	0.71	0.70	0.51
31. Develop potential and pursue interests	0.65	0.77	0.65
14. Plan what happens on weekends	0.65	0.65	0.45
30. Support to make important decisions about their life	0.64	0.69	0.49
32. Support to live the life they want	0.63	0.75	0.59
13. When and where go out is based on preferences	0.62	0.62	0.39
8. Decisions made with the residents	0.57	0.58	0.37
20. Opportunities and support to make everyday choices	0.57	0.68	0.48
29. Find ways to involve in activities they enjoy	0.56	0.72	0.59
28. Meet people and make friends	0.56	0.67	0.49
24. When cooking or cleaning, residents are involved	0.53	0.61	0.40
23. Take part in household tasks on a daily basis	0.50	0.60	0.42
Factor 2: Factional (*α* = 0.90; GLB = 0.94)			
77. Distinct groups of staff, rather than one staff team^a^	0.77	0.82	0.70
75. Close relationships amongst staff negative impact^a^	0.77	0.78	0.61
76. Some staff have too much influence^a^	0.76	0.77	0.60
74. Some staff do not cooperate^a^	0.73	0.73	0.55
78. Some staff do not follow the HS’s directions^a^	0.71	0.76	0.61
73. Some staff do not follow policy and procedures^a^	0.56	0.62	0.41
81. House Supervisor has difficulty managing some staff^a^	0.50	0.62	0.50
80. Conflict between the HS and some staff^a^	0.45	0.57	0.46
Factor 3: Effective team leadership (*α* = 0.92; GLB = 0.94)			
83. House supervisor role models	−0.85	−0.85	0.73
85. House supervisor positive influence	−0.83	−0.89	0.80
84. House supervisor teaches staff better ways to support	−0.82	−0.86	0.76
86. House supervisor acknowledges when staff work well	−0.75	−0.80	0.65
82. House supervisor explains to staff what the aims are	−0.73	−0.76	0.60
Factor 4: Collaboration within the organization (*α* = 0.85; GLB = 0.88)			
61. SM understand what it is like to work here	0.79	0.79	0.64
63. SM help us to find solutions to problems	0.77	0.78	0.62
62. Communication between staff and SM	0.77	0.79	0.65
64. Complain about the priorities of this organization^a^	0.57	0.64	0.48
65. Conflict about how residents are supported^a^	0.48	0.57	0.42
66. Critical of the organization^a^	0.46	0.51	0.32
Factor 5: Valuing residents and relationships (*α* = 0.88; GLB = 0.92)			
43. Value relationships	0.75	0.78	0.62
42. Talk about things that are of interest	0.72	0.73	0.54
41. Take an interest in residents’ lives	0.70	0.79	0.64
46. Celebrate when achieve something important	0.66	0.70	0.52
40. Enjoy spending time	0.61	0.74	0.60
45. Have fun together	0.59	0.66	0.46
48. Try new experiences we think they will enjoy	0.42	0.56	0.36
Factor 6: Social distance from residents (*α* = 0.86; GLB = 0.87)			
37. Believe like children^a^	0.88	0.85	0.74
38. Believe different to the residents^a^	0.77	0.76	0.58
35. Talk like they are talking to children^a^	0.65	0.76	0.63
39. Believe will never participate in the community^a^	0.57	0.71	0.60
36. Mimic residents^a^	0.54	0.61	0.39
Factor 7: Alignment of staff with organizational values (*α* = 0.81; GLB = 0.88)			
71. Values match the organization's values	−0.71	−0.80	0.68
70. We share similar values	−0.59	−0.67	0.56
67. Mission and values understood	−0.51	−0.61	0.44
68. Values guide staff support	−0.47	−0.62	0.55
72. Purpose and priorities understood	−0.46	−0.59	0.43

*N* = 343.

Abbreviations:* h* ^2^ = communality; GLB, greatest lower bound; HS, House Supervisor; SM, Senior Managers.

a Reverse scored. Items in the table have been abbreviated. Item numbers are from the questionnaire.

**Table 4:** Summary statistics, intercorrelations and sum of squared structure loadings for the Group Home Culture Scale (*N* = 343)

Factor	*M*	*SD*	1	2	3	4	5	6	7
1. Supporting well‐being	3.93	0.57	–						
2. Factional^a^	3.48	0.82	0.22	–					
3. Effective team leadership	4.03	0.77	−0.31	−0.28	–				
4. Collaboration within the organization	3.27	0.80	0.30	0.28	−0.22	–			
5. Valuing residents and relationships	4.28	0.48	0.47	0.14	−0.34	0.13	–		
6. Social distance from residents^a^	4.10	0.73	0.42	0.28	−0.21	0.23	0.42	–	
7. Alignment of staff with organizational values	3.91	0.56	−0.36	−0.25	0.24	−0.33	−0.31	−0.29	–
Sum of squared structure loadings			9.37	6.19	6.48	5.36	7.69	6.60	5.46

a Factors are reverse scored.

## 4 DISCUSSION

This study has resulted in the development of an instrument to measure dimensions of organizational culture in group homes, named the Group Home Culture Scale (GHCS). It contrasts to the generic instruments used in previous research of intellectual disability services (Gillett & Stenfert‐Kroese, 2003; Hatton et al., 1999) in having direct relevance to group homes. Further, the GHCS was found to have acceptable content validity, to be acceptable to people experienced in working in group homes, and each of the seven dimensions derived through the EFA demonstrated very good internal consistency. In these ways, the GHCS was found to meet recommended criteria for scale development (DeVellis, 2012; Wymer & Alves, 2013).

The GHCS provides a means to measure many of the key characteristics of group home culture identified by Bigby and colleagues (Bigby & Beadle‐Brown, 2016; Bigby, Knox, et al., 2012), but with more refined dimensions compared to those proposed. The number of dimensions was expanded from five to seven as some of the content domains of the original proposed dimensions were made more salient by being measurable on separate subscales. During the item generation stage, when each of the original dimensions was defined and the content domains were specified (DeVellis, 2012; Hinkin, 1998; Wymer & Alves, 2013), each of the original dimensions was found to comprise several content domains. For example, the original dimension alignment of power holders’ values comprised several content domains, which manifested in different ways. In four of the five underperforming group homes, the values of the power holders (which may or may not have included the front‐line supervisors) were misaligned with those espoused by the organization (Bigby, Knox, et al., 2012). On the other hand, in another underperforming group home, power was more dispersed, with each staff member adopting his or her own way of working (Bigby, Knox, et al., 2012). In contrast, Bigby and Beadle‐Brown (2016) found that in each of the better performing group homes, the formally appointed power holder—the front‐line supervisor—influenced the staff team, and his or her values aligned with the organization's espoused values. Furthermore, staff shared common values that aligned with the organization's espoused values.

Given the complexity of this dimension and the different ways it manifested in the group homes, it was difficult to write concise items that effectively tapped it, while also adhering to the item development guideline that each item convey a single idea (i.e. not double‐barrelled; DeVellis, 2012). The approach adopted was to write a number of items, and the EFA showed that they formed four distinct factors: Factional, Effective Team Leadership, Collaboration within the Organization and Alignment of Staff with Organizational Values.

Similarly, some characteristics of group home culture that were identified by Bigby and colleagues (Bigby & Beadle‐Brown, 2016; Bigby, Knox, et al., 2012), but were not central to the conceptualization of the original dimensions, have been made more salient by being the focal construct measured on a subscale. For example, in their description of the original dimension of orientation to change and new ideas, Bigby, Knox, et al. (2012) noted how staff in the underperforming group homes felt distanced from senior managers and the broader organization. Although staff members’ perceptions of the organization and senior managers were part of the description for this dimension, it was not central to its conceptualization. Nonetheless, some items written to tap staff members’ perception of the organization and of senior managers, along with others written to tap the dimension alignment of power holders’ values, were found to comprise a common underlying dimension, which was named Collaboration within the Organization. This newly identified dimension refers to the extent to which staff have a positive perception of organizational support and priorities, that is their orientation to the organization.

The development of the GHCS also showed overlapping content domains across some of the original dimensions proposed by Bigby and colleagues (Bigby & Beadle‐Brown, 2016; Bigby, Knox, et al., 2012). For example, the original dimensions of perceived purpose and working practices differed conceptually in that perceived purpose was essentially about the way staff thought about their role, whereas working practices was about the way staff performed their role. However, during the item development stage, when the content domains of these two dimensions were specified, the way they manifested in terms of how staff conducted their shifts and supported residents were found to be similar. Items developed to tap each of these dimensions were shown through the EFA to form a common factor—Supporting Well‐Being—indicating that they reflect the same underlying dimension of group home culture.

An unexpected result was the failure of the GHCS to include as a dimension staff resistance or openness to change and new ideas (Bigby & Beadle‐Brown, 2016; Bigby, Knox, et al., 2012). This result may be explained by small intercorrelations and factor loadings among some of the items written to tap this content domain, indicating that they did not comprise a common underlying dimension of group home culture. In part, problems with these items could reflect difficulties for respondents in rating them consistently because how staff respond to change and new ideas can also be inconsistent. Bigby, Knox, et al. (2012) characterized the culture in the underperforming group homes as resistance to change and new ideas, but some of their evidence suggested that some staff, rather than all, were exhibiting resistance. On the other hand, Bigby and Beadle‐Brown (2016) characterized the culture in the better performing group homes as openness to change and new ideas, but, as they noted, a family member's suggestion about how to decorate a resident's room was opposed by a staff member because it was thought to be inconsistent with the resident's preferences. It would appear, then, that context is a potential factor when considering how staff respond to new ideas.

A limitation of this study was that the GHCS was developed based on research of underperforming (Bigby, Knox, et al., 2012) and better performing group homes (Bigby & Beadle‐Brown, 2016; Bigby et al., 2015), instead of those considered to be of high quality. In general, there has been a lack of research into culture in high‐quality group homes, and rather, more is known about the culture in poor quality and abusive services (see Bigby, Knox, et al., 2012; Cambridge, 1999; Hutchison & Stenfert‐Kroese, 2015; Marsland et al., 2007). To enhance the GHCS, new items could be generated that better reflect the culture in high‐quality services; however, qualitative research that explicates the characteristics of these services may first be required. Another limitation of this study was that the level of adaptive behaviour of the people who lived in the group homes was not measured, which meant that the potential effect of resident level of adaptive behaviour on staff member ratings of GHCS items was not assessed. Future research is needed to determine any potential relationship between resident adaptive behaviour and GHCS scores.

Of relevance to practice is that the GHCS can be used by organizations to measure staff perceptions of their work culture. Information collected with the GHCS has potential to be used by organizations to understand culture in group homes and identify opportunities to improve it. Potential advantages of using the GHCS in group homes over a generic instrument is that the findings could have clearer implications for service delivery and be more translatable into strategies to change or maintain culture.

With the use of the GHCS, there is the potential for future research into identifying the dimensions of organizational culture that are associated with the quality of staff support and QOL outcomes for people with intellectual disabilities who live in group homes. The GHCS also has potential use in research to examine whether dimensions of group home culture are associated with staff outcomes, such as job satisfaction. To test the factor structure of the GHCS, further research is needed using confirmatory factor analysis.

## APPENDIX 1

**Table A1:** Pattern matrix for the Group Home Culture Scale

Item	Factor loading						
	1	2	3	4	5	6	7
Factor 1: Supporting well‐being							
16. Involve in their local community	**0.71**	−0.07	−0.06	0.03	−0.03	−0.06	−0.06
31. Develop potential and pursue interests	**0.65**	0.06	0.07	−0.07	0.19	0.05	−0.10
14. Plan what happens on weekends	**0.65**	0.10	−0.06	−0.04	−0.09	0.08	0.02
30. Support to make important decisions about their life	**0.64**	0.02	−0.05	−0.04	0.10	0.01	0.02
32. Support to live the life they want	**0.63**	0.04	−0.01	0.04	0.05	0.09	−0.08
13. When and where go out is based on preferences	**0.62**	0.04	−0.01	0.05	−0.05	0.00	0.01
8. Decisions made with the residents	**0.57**	0.07	−0.12	−0.05	−0.14	0.08	−0.01
20. Opportunities and support to make everyday choices	**0.57**	−0.00	−0.04	0.07	0.10	0.01	−0.06
29. Find ways to involve in activities they enjoy	**0.56**	0.00	0.05	0.02	0.28	−0.04	−0.13
28. Meet people and make friends	**0.56**	−0.03	0.03	0.07	0.21	−0.04	−0.06
24. When cooking or cleaning, residents are involved	**0.53**	−0.00	0.04	0.09	0.09	0.09	0.05
23. Take part in household tasks on a daily basis	**0.50**	−0.01	0.03	0.01	0.20	0.14	0.12
Factor 2: Factional							
77. Distinct groups of staff, rather than one staff team^a^	−0.01	**0.77**	−0.02	0.03	0.12	0.01	−0.08
75. Close relationships amongst staff negative impact^a^	−0.10	**0.77**	0.04	0.06	0.04	0.04	−0.03
76. Some staff have too much influence^a^	−0.06	**0.76**	−0.06	−0.03	0.01	0.06	0.02
74. Some staff do not cooperate^a^	0.16	**0.73**	0.09	0.04	−0.02	−0.09	−0.02
78. Some staff do not follow the HS’s directions^a^	0.11	**0.71**	0.01	0.06	0.01	−0.00	−0.07
73. Some staff do not follow policy and procedures^a^	0.07	**0.56**	0.04	0.03	−0.05	0.08	−0.12
81. House Supervisor has difficulty managing some staff^a^	0.10	**0.50**	−0.30	0.07	−0.01	−0.00	0.00
80. Conflict between the HS and some staff^a^	0.07	**0.45**	−0.34	0.07	−0.03	−0.03	−0.01
Factor 3: Effective team leadership							
83. House supervisor role models	0.04	−0.02	−**0.84**	−0.02	−0.03	0.04	−0.04
85. House supervisor positive influence	0.04	0.10	−**0.83**	0.01	0.05	−0.01	0.02
84. House supervisor teaches staff better ways to support	−0.01	−0.05	−**0.82**	0.05	0.07	0.02	−0.07
86. HS acknowledges when staff work well	0.04	−0.01	−**0.75**	0.02	0.09	0.01	−0.00
82. HS explains to staff what the aims are	0.01	−0.08	−**0.73**	0.10	0.05	0.04	0.00
Factor 4: Collaboration within the organization							
61. SM understand what it is like to work here	−0.02	−0.04	−0.08	**0.79**	−0.00	−0.04	−0.04
63. SM help us to find solutions to problems	0.04	−0.06	−0.04	**0.77**	0.03	−0.07	−0.05
62. Communication between staff and SM	0.05	−0.05	−0.08	**0.77**	−0.05	−0.07	−0.10
64. Complain about the priorities of this organization^a^	0.02	0.24	0.09	**0.57**	−0.05	0.08	−0.03
65. Conflict about how residents are supported^a^	0.01	0.24	−0.06	**0.48**	0.03	0.12	0.07
66. Critical of the organization^a^	−0.06	0.15	−0.04	**0.46**	0.06	0.15	0.07
Factor 5: Valuing residents and relationships							
43. Value relationships	−0.04	0.02	0.02	−0.00	**0.75**	0.11	−0.02
42. Talk about things that are of interest	−0.04	0.04	−0.01	0.01	**0.72**	0.07	0.03
41. Take an interest in residents’ lives	0.06	0.03	0.00	−0.03	**0.70**	0.06	−0.11
46. Celebrate when achieve something important	0.10	0.02	−0.10	0.01	**0.66**	−0.03	0.10
40. Enjoy spending time	0.12	−0.04	−0.03	0.08	**0.61**	0.05	−0.11
45. Have fun together	0.03	−0.01	−0.12	0.01	**0.59**	0.00	−0.06
48. Try new experiences we think they will enjoy	0.15	−0.02	−0.12	−0.05	**0.42**	0.01	−0.10
Factor 6: Social distance from residents							
37. Believe like children^a^	0.04	−0.08	−0.05	−0.03	−0.06	**0.88**	0.00
38. Believe different to the residents^a^	−0.01	−0.05	−0.02	−0.02	0.01	**0.77**	−0.03
35. Talk like they are talking to children^a^	0.05	0.14	−0.05	−0.07	0.08	**0.65**	−0.08
39. Believe will never participate in the community^a^	0.23	−0.10	0.07	0.08	0.13	**0.57**	−0.04
36. Mimic residents^a^	−0.00	0.08	0.00	0.06	0.08	**0.54**	−0.01
Factor 7: Alignment of staff with organizational values							
71. Values match the organization's values	−0.02	0.17	−0.03	0.02	0.08	0.04	**−0.71**
70. We share similar values	−0.05	0.21	−0.18	−0.15	0.09	0.07	**−0.59**
67. Mission and values understood	0.11	−0.07	0.02	0.22	−0.09	0.12	**−0.51**
68. Values guide staff support	0.19	−0.16	0.06	0.36	0.03	0.04	**−0.47**
72. Purpose and priorities understood	0.02	0.12	−0.07	0.04	0.16	0.05	**−0.46**

Abbreviations: HS, House Supervisor; SM, Senior Managers.

a Reverse scored. Items in the table have been abbreviated. Loadings highlighted in bold indicate the factor on which the item was placed.

**Table A2:** Structure matrix for the Group Home Culture Scale

Item	Factor loading						
	1	2	3	4	5	6	7
Factor 1: Supporting well‐being							
31. Develop potential and pursue interests	**0.77**	0.23	−0.23	0.19	*0.53*	*0.42*	−0.38
32. Support to live the life they want	**0.75**	0.25	−0.28	0.30	*0.43*	*0.43*	−0.38
29. Find ways to involve in activities they enjoy	**0.72**	0.18	−0.24	0.25	*0.56*	0.34	−*0.40*
16. Involve in their local community	**0.70**	0.11	−0.26	0.24	0.31	0.25	−0.30
30. Support to make important decisions about their life	**0.69**	0.18	−0.27	0.17	0.*41*	0.32	−0.24
20. Opportunities and support to make everyday choices	**0.68**	0.19	−0.28	0.29	*0.42*	0.34	−0.33
28. Meet people and make friends	**0.67**	0.13	−0.22	0.26	*0.47*	0.30	−0.32
14. Plan what happens on weekends	**0.65**	0.25	−0.25	0.19	0.27	0.34	−0.23
13. When and where go out is based on preferences	**0.62**	0.19	−0.21	0.24	0.26	0.27	−0.23
24. When cooking or cleaning, residents are involved	**0.61**	0.16	−0.18	0.26	0.36	0.35	−0.22
23. Take part in household tasks on a daily basis	**0.60**	0.13	−0.20	0.17	*0.45*	0.39	−0.16
8. Decisions made with the residents	**0.58**	0.22	−0.27	0.17	0.21	0.30	−0.22
Factor 2: Factional							
77. Distinct groups of staff, rather than one staff team^a^	0.26	**0.82**	−0.30	0.29	0.26	0.30	−0.32
75. Close relationships amongst staff negative impact^a^	0.12	**0.78**	−0.19	0.26	0.13	0.24	−0.22
76. Some staff have too much influence^a^	0.14	**0.77**	−0.26	0.19	0.13	0.25	−0.17
78. Some staff do not follow the HS’s directions^a^	0.31	**0.76**	−0.25	0.31	0.19	0.28	−0.30
74. Some staff do not cooperate^a^	0.27	**0.73**	−0.15	0.26	0.11	0.17	−0.22
81. House Supervisor has difficulty managing some staff^a^	0.31	**0.62**	−*0.48*	0.30	0.22	0.25	−0.24
73. Some staff do not follow policy and procedures^a^	0.24	**0.62**	−0.17	0.24	0.12	0.27	−0.28
80. Conflict between the HS and some staff^a^	0.27	**0.57**	−*0.49*	0.29	0.19	0.20	−0.23
Factor 3: Effective team leadership							
85. House supervisor positive influence	0.33	0.35	**−0.89**	0.23	0.36	0.23	−0.23
84. House supervisor teaches staff better ways to support	0.31	0.23	**−0.86**	0.25	0.37	0.24	−0.29
83. House supervisor role models	0.30	0.23	**−0.85**	0.19	0.30	0.23	−0.24
86. HS acknowledges when staff work well	0.32	0.23	**−0.80**	0.21	0.37	0.23	−0.23
82. HS explains to staff what the aims are	0.29	0.18	**−0.76**	0.26	0.32	0.22	−0.22
Factor 4: Collaboration within the organization							
62. Communication between staff and SM	0.27	0.19	−0.25	**0.79**	0.10	0.13	−0.33
61. SM understand what it is like to work here	0.23	0.20	−0.24	**0.79**	0.11	0.15	−0.29
63. SM help us to find solutions to problems	0.28	0.17	−0.22	**0.78**	0.15	0.14	−0.30
64. Complain about the priorities of this organization^a^	0.24	*0.40*	−0.12	**0.64**	0.09	0.25	−0.27
65. Conflict about how residents are supported^a^	0.27	*0.41*	−0.26	**0.57**	0.19	0.31	−0.21
66. Critical of the organization^a^	0.19	0.31	−0.20	**0.51**	0.17	0.28	−0.17
Factor 5: Valuing residents and relationships							
41. Take an interest in residents’ lives	*0.45*	0.18	−0.30	0.14	**0.79**	*0.41*	−0.37
43. Value relationships	0.37	0.15	−0.26	0.12	**0.78**	*0.41*	−0.28
40. Enjoy spending time	*0.50*	0.15	−0.32	0.24	**0.74**	*0.40*	−0.39
42. Talk about things that are of interest	0.34	0.15	−0.26	0.12	**0.73**	0.36	−0.22
46. Celebrate when achieve something important	*0.40*	0.13	−0.33	0.11	**0.70**	0.29	−0.16
45. Have fun together	0.37	0.14	−0.35	0.14	**0.66**	0.30	−0.28
48. Try new experiences we think they will enjoy	*0.41*	0.11	−0.32	0.10	**0.56**	0.29	−0.29
Factor 6: Social distance from residents							
37. Believe like children^a^	0.37	0.17	−0.20	0.17	0.32	**0.85**	−0.23
35. Talk like they are talking to children^a^	*0.42*	0.36	−0.28	0.19	*0.43*	**0.76**	−0.34
38. Believe different to the residents^a^	0.31	0.17	−0.17	0.15	0.33	**0.76**	−0.24
39. Believe will never participate in the community^a^	*0.53*	0.14	−0.16	0.27	*0.47*	**0.71**	−0.32
36. Mimic residents^a^	0.30	0.26	−0.18	0.21	0.32	**0.61**	−0.23
Factor 7: Alignment of staff with organizational values							
71. Values match the organization's values	0.34	0.38	−0.28	0.32	0.34	0.33	**−0.80**
70. We share similar values	0.29	0.39	−0.37	0.15	0.35	0.32	**−0.67**
68. Values guide staff support	*0.44*	0.09	−0.16	*0.52*	0.28	0.30	**−0.62**
67. Mission and values understood	0.35	0.16	−0.16	*0.41*	0.19	0.30	**−0.61**
72. Purpose and priorities understood	0.35	0.31	−0.30	0.28	0.38	0.32	**−0.59**

Abbreviations: HS, House Supervisor; SM, Senior Managers.

a Reverse scored. Items in the table have been abbreviated. Loadings highlighted in bold indicate the factor on which the item was placed. Italicized values indicate cross‐loading.
